# Public health round-up

**DOI:** 10.2471/BLT.16.010616

**Published:** 2016-06-01

**Air pollution in cities**

A woman with respiratory illness on a bus in New Delhi, India. Some 615 cities with a population greater than 100 000 in low- and middle-income countries report air quality data to the World Health Organization (WHO). About 98% or 603 of those cities – including New Delhi – do not meet WHO air quality standards.

**Figure:**
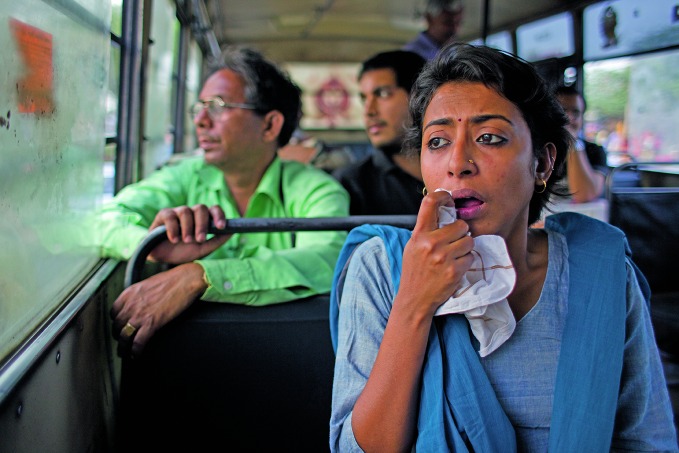
WHO /Diego Rodriguez

## New process for DG election

The process to elect a new Director-General of the World Health Organization (WHO) has started with a new code of conduct, new forums for candidates to interact with Member States and a new electronic voting system to promote transparency and fairness. 

The race began on 22 April, when WHO’s 194 Member States were invited to propose candidates. Their proposals will be made public on 23 September. 

In October, officials from Member States will be given the opportunity to ask the candidates questions and have virtual discussions with them in a protected web forum hosted on the WHO website. It will be broadcast through a protected website for representatives of Member States and Associate Members who cannot attend.

The candidates will then be invited to present their vision in person to representatives of WHO Member States and Associate Members and answer questions during a candidates’ forum of a maximum of three days in November held at WHO headquarters. 

Using a new electronic voting system, WHO’s Executive Board will draw up a short list of up to five candidates in January 2017 based on one or more secret ballots. 

Members of the Executive Board will then interview these candidates and nominate up to three of them to go forward to the World Health Assembly (WHA) in May 2017 for the final round of voting. Previously, only one nomination was submitted by WHO’s Executive Board to the World Health Assembly. 

The WHA will consider the Executive Board’s nomination at a private meeting and come to a decision by secret ballot.

Dr Margaret Chan, who was elected Director-General in 2006, will complete her second term on 30 June 2017 and the new Director-General will take office the next day. 

The new code of conduct guides the behaviour and roles of Member States, candidates and the WHO Secretariat during the electoral process. It calls for openness, dignity, equity and good faith, as well as financial disclosure and avoidance of conflicts of interest.

http://apps.who.int/gb/ep/

## Tougher action needed on breast-milk substitutes

Breast-milk substitutes are still being marketed too aggressively in many countries, 35 years after an international code was adopted to promote breastfeeding and, in turn, to protect infant health. 

A new report, released by WHO, the United Nations Children’s Fund and the International Baby Food Action Network last month, reveals major gaps in the implementation of the International Code of Marketing of Breast-milk Substitutes.

Only 39 of the 194 countries analysed had legislation as of March 2016 incorporating all or most of the Code’s provisions, a slight increase from 37 in 2011, the new report showed. 

The Code calls on countries to protect breastfeeding by stopping the inappropriate marketing of breast-milk substitutes (including infant formula) and free distribution of feeding bottles and teats.

But the report, entitled *Marketing of breast-milk substitutes: national implementation of the international Code, status report 2016*, found that only 66 of these countries prohibit the advertising of breast-milk substitutes and 64 prohibit the provision of free samples. 

It found that 49 of these countries prohibit free or low-cost supplies of breast-milk substitutes to health centres, while 60 prohibit gifts of any kind from relevant manufacturers to health workers.

WHO and UNICEF recommend that mothers exclusively breastfeed infants for the child's first six months to achieve optimal growth, development and health. 

After that, mothers should continue breastfeeding and feed their children other safe and nutritionally adequate foods until two years of age or beyond.

To support these recommendations, WHO’s 194 Member States have committed to increasing the rate of exclusive breastfeeding in the first six months of life to at least 50% by 2025, as one of a set of global nutrition targets.

Annual sales of breast-milk substitutes amounted to US$ 44.8 billion worldwide in 2014, according to a study published in the *Lancet* in January. 

http://www.who.int/nutrition/publications/infantfeeding/code_report2016/

## Shorter drug regimens for MDR–TB

New WHO recommendations aim to speed up detection and improve treatment outcomes for multidrug-resistant tuberculosis (MDR–TB) through use of a novel rapid diagnostic test and a shorter, cheaper treatment regimen.

“This is a critical step forward in tackling the MDR–TB public health crisis,” said Dr Mario Raviglione, Director of WHO’s Global TB Programme. 

“The new WHO recommendations offer hope to hundreds of thousands of MDR–TB patients who can now benefit from a test that quickly identifies eligibility for the shorter regimen, and then complete treatment in half the time and at nearly half the cost.”

The new treatment regimen costs less than US$ 1000 per patient and can be completed in 9–12 months. The conventional treatment regimens, which take 18–24 months to complete, yield low cure rates: just 50% on average globally. 

The shorter MDR–TB regimen is expected to improve outcomes and potentially decrease deaths due to better adherence to treatment and reduced loss to follow-up because of the shorter treatment duration. 

This regimen is recommended for MDR-TB patients (adults and children) who do not have resistance to fluoroquinolones or second line injectable agents, the two most effective components of the regimen. 

It is also recommended for individuals who have not yet been treated with second-line drugs and people living with HIV. 

http://www.who.int/tb/areas-of-work/drug-resistant-tb/treatment/resources/

**Cover photo**

**Syrians evacuate an injured man amid the rubble of destroyed buildings in the northern Syrian city of Aleppo last year, following bombardments that severely damaged a local health centre. Last month the city’s Al Quds Hospital was destroyed in an attack that killed dozens of children, patients and other health workers. The Al Quds Hospital was supported by the International Committee of the Red Cross (ICRC) and Médecins Sans Frontières (MSF). The ICRC is leading a campaign with partners including MSF and the World Health Organization (WHO), called Health Care in Danger to address the issue of violence against patients, health-care workers, facilities and vehicles, and to ensure that health workers can safely deliver health care in armed conflicts and other emergencies. http://healthcareindanger.org/hcid-project/**

**Figure:**
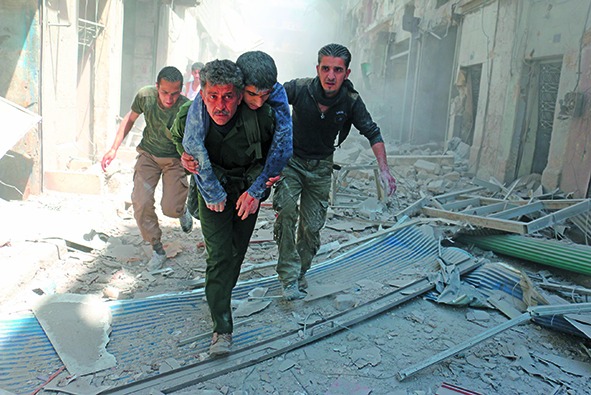
Ameer Alhalbi/AFP/Getty Images

## Guidelines on FGM management

WHO released new guidelines last month on the management of health complications from female genital mutilation (FGM), defined as any procedure that intentionally alters or causes injury to the female genital organs for non-medical reasons. 

The guidelines stress that women and girls who have been subjected to FGM “have experienced a harmful practice and should be provided quality health care” and that all efforts must be made to prevent FGM. 

More than 200 million girls and women globally have undergone FGM. Most live in 30 countries in the WHO African, Eastern Mediterranean and South-East Asia regions. Many girls and women living in diaspora populations, including in Europe and North America, have also undergone the practice or may be at risk of being pressured into undergoing FGM. 

FGM may have devastating physical, psychological, and social consequences for the women affected and is a widespread problem. 

The guidelines condemn the growing trend of the medicalization of FGM – when FGM is performed by health-care providers – and recommend several approaches to repairing physical damage caused by FGM as well as recommendations on managing the mental health and female reproductive health consequences of FGM. 

The guidelines, launched at the Women Deliver Conference in Copenhagen on 16–19 May, are intended as a basis that can be used to develop local and national guidelines and that can be integrated into training programmes for health professionals.

www.who.int/reproductivehealth/topics/fgm/management-health-complications-fgm/en/

## Paediatric emergency guideline updated

WHO has released an updated guideline on what to do when very sick children arrive at a hospital in low-resource settings. 

The updated *Paediatric emergency triage, assessment and treatment: care of critically-ill children guideline* covers the most common emergency conditions in these children, including breathing problems, circulatory impairment or shock, coma or convulsive seizures, and severe dehydration.

The recommendations in this publication complement or update guidance published in the Emergency Triage Assessment and Treatment (ETAT) training materials published in 2005 and the second edition *Pocket book of hospital care for children* published in 2013. 

The updated guideline does not cover all WHO recommendations on paediatric ETAT, but only those identified by the WHO guideline development group in 2013. 

The guideline includes recommendations on oxygen therapy, the management of children with impaired circulation or shock with intravenous fluids, and treatment of children with acute seizures with anticonvulsant medicines when intravenous access is or is not available.

http://www.who.int/maternal_child_adolescent/documents/paediatric-emergency-triage-update/en/

**Looking ahead**
**23–28 May – Sixty-ninth World Health Assembly, Geneva, Switzerland**
**31 May – World No Tobacco Day**
**14 June – World Blood Donor Day**

